# Identification of microalgae cultured in Bold’s Basal medium from freshwater samples, from a high-rise city

**DOI:** 10.1038/s41598-021-84112-0

**Published:** 2021-02-24

**Authors:** Charmaine Lloyd, Kai Heng Tan, Kar Leong Lim, Vimala Gana Valu, Sarah Mei Ying Fun, Teng Rong Chye, Hui Min Mak, Wei Xiong Sim, Sarah Liyana Musa, Joscelyn Jun Quan Ng, Nazurah Syazana Bte Nordin, Nurhazlyn Bte Md Aidzil, Zephyr Yu Wen Eng, Punithavathy Manickavasagam, Jen Yan New

**Affiliations:** 1 School of Life Sciences and Chemical Technology - Microalgal Research Group, Ngee Ann Polytechnic, Clementi, Singapore; 2 School of Health and Medical Sciences, Swinburne University of Technology, Melbourne, VIC 3122 Australia

**Keywords:** Ecology, Microbiology, Microbial communities, Industrial microbiology

## Abstract

This study aimed at exploring microalgal heterogeneity from fresh water samples collected from inland water bodies in the heavily built city of Singapore. Culturable pure isolates (n = 94) were subject to an in-house microalgal DNA extraction method and LSU rDNA sequencing. Isolates were analysed for their predominance and distribution. A total of 17 different algal genera were identified (H = 2.8, E_H_ = 0.6), of which *Scenedesmus* spp. and *Chlorella* spp. constituted 27.5% and 21.3% of isolates respectively, followed by *Micractinium* spp. (18.8%) and *Chlamydomonas* spp. (12.5%). We also report 16 new microalgal strains from this region. The data is important from an ecological and biotechnological perspective.

## Introduction

Algae are viewed as potential sources of biodiesel, nutraceuticals, cosmetics, pharmaceuticals, fertilizers and food sources for human and animal consumption^1^. Exploratory studies on microalgal communities in various natural ecosystems provide knowledge that is both of ecological and commercial value^2–4^. Anthropogenic stress may play a role in defining microbial ecosystems^5^ and is thus worth exploring. The city-state island of Singapore is approximately 722.5 square km. It comprises around 10,099 high-rise buildings and is highly populated (5.7 million)^6^. The island is interspersed with several artificial canals and drains which meander through, to facilitate rain water collection^7^. Past studies have focussed on marine and macroalgae in the South East Asian region^2,3^. The aim of our study was to identify culturable microalgae from water samples collected from random sampling sites.

As with most eukaryotes, microalgae have different morphologies during their life cycle. Some exhibit phenotypic plasticity in the same culture as well. Microscopic identification depends largely on expertise in phycology and may not be a reliable way of identifying isolates. Hence, we aimed to use LSUr (large subunit ribosomal) DNA sequencing^8^ to help facilitate the identification process. The extremely hardy nature of microalgal cell walls^9,10^ is relatively resistant to various hydrolysis methods used to extract nuclear content. Methods used to digest the cell wall often require to be adapted from other protocols, to suit the algal genus researched on. There are reports of success with using commercial DNA extraction kits^11,12^, phenol–chloroform methods^13^, glass bead disruption, Tris–HCl EDTA-NaCl-SDS extraction^14^, hexadecyl-trimethyl-ammonium bromide (CTAB)^15,16^, freeze thaw techniques, tissue homogenizing, grinding^17^ and boiling^18^. We modified an existing method^14^ to facilitate DNA extraction of our algal isolates.

## Materials and methods

### Isolation and culture

Surface water samples were collected in sterile containers from publicly accessible inland water locations (n = 31) in the north (NZ)-6, central (CZ)-11, east (EZ)-6 and West zones (WZ)-8, by random sampling. Samples were pelleted, washed, resuspended in sterile Bold’s basal medium (BBM) broth and serially diluted until clear. A loopful from the last three clear dilutions were examined microscopically for the presence of algae and cultured using spread-plate onto BBM agar. Triplicate isolation plates were incubated in a 23 °C room with a tube light source for 24 h. Swarming contamination was controlled using 2–2.5% agar. Isolates were sub-cultured until pure. Pure colonies were stored in 15% glycerol-BBM at − 20 °C for long-term and in BBM slants for short-term stocks.

### DNA extraction, PCR and sequencing

DNA was extracted from pure isolates by a slight modification of the protocol of Martin-Laurent *et al*^14^. Briefly 5–6 microalgal pure colonies were added to 500 µl of lysis buffer [(100 mM Tris-HCl pH8, 100 mM EDTA, 100 mM NaCl, 1% (w/v) Polyvinylpyrrolidone, 2%(w/v) sodium dodecyl sulphate)] in a microcentrifuge tube. The tube was dipped in liquid nitrogen for 5 s (4 times) and sonicated at 7 watts for 6 s (5 times). A volume of 500 µl of phenol:chloroform:isoamylalcohol (25:24:1) was added to the suspension and then centrifuged at 14,000 rpm for 5 min. The supernatant was combined with 1/10 volume of 5 M sodium acetate and placed on ice for 10 min, after which it was centrifuged at 14,000 rpm for 5 min. One volume of ice-cold 100% ethanol was added and DNA pelleted by centrifugation (13,000 rpm for 10 min, 4 °C). The pellet was air-dried, and the DNA was stored in 50 µl of TE buffer. The PCR reaction mixture was performed in a total volume of 50 µl. Five microliter of genomic DNA in TE buffer pH 8.0, 1 µl each of forward and reverse primer, 25 µl of PCR master mix (Promega, P119A, USA) and 18 µl of nuclease free water. The protocol for PCR amplification was initial denaturation (94 °C, 3 min, 1 cycle), 35 cycles of denaturation (95 °C, 45 s), annealing (47.5 °C, 1 min) and extension (72 °C, 1.3 min); followed by final extension (72 °C, 5 min, 1 cycle). The D1-D2 LSU rDNA sequences for each microalga was amplified with universal primers^19^. On agarose gel electrophoresis, bands in between 200 and 1000 bp were excised and purified using a gel extraction kit. The PCR products were outsourced to AIT Biotech Company Singapore for DNA sequencing services.

### Analysis

The LSU rDNA sequences obtained were blasted against available sequences from GenBank data base for identification. Shannon diversity index (*H*) and Evenness (E_H_) was calculated as a measure of diversity of both genera and species in the locations and zones. Menhinick’s index (D) was calculated for the number of species per zone^20^. Poisson regression and Poisson regression allowing for over-dispersion was used to analyse whether significant associations could be made between zones, locations and identified strains.

## Results

With LSU rDNA sequencing, a total of 94 cultivable isolates could be identified. Fourteen isolates which were found to be similar in species identity for particular locations were not included in subsequent analysis. Eighty isolates were further analysed (Table 1). Seventeen different genera could be identified among the 80 isolates (*H* = 2.8, E_H_ = 0.6). The most common genera across the 80 isolates were *Scenedesmus* spp. (27.5%), *Chlorella* spp. (18.8%), *Micractinium* spp. (18.8%) and *Chlamydomonas* spp. (12.5%) (Table 1). As seen in the table, several species of the three major genera were observed in our collection. From Table 2, genus richness (D) was slightly higher for CZ(4.5) followed by WZ(3.7), EZ(3.3) and least in the NZ(2.7). When compared for uni and multi algal heterogeneity in each sample, 7 locations had three or more genera. Two locations CL (in the WZ) and location BG (CZ) had 6 genera. CL and BG also had higher strain diversity in our study (*H* = 1.7), followed by CG (*H* = 1.5) and USR, BG (*H* = 1.29). Zone and location associations with strains in this study were not found to be significant.

**Table 1:** Microalgal diversity among LSU-rDNA sequenced microalgae and their distribution.

Microalgal genera	Species (25)	Microalgal species—strain level (80)	Zone distribution of species			
			CZ	NZ	EZ	WZ
			26	16	9	29
*Scenedesmus spp.* (22)	1	*Scenedesmus obliquus YSW14*	4	1	1	2
		*Scenedesmus obliquus YSR02*	1			
		*Scenedesmus obliquus YSW17*				1
		*Scenedesmus obliquus*	3	1		1
	2	*Scenedesmus pectinatus*				2
	3	*Scenedesmus acuminatus*				1
	4	*Scenedesmus acutus*				1
	5	*Scenedesmus bajacalifornicus*				1
	u	*Scenedesmus sp (unidentified)*			1	1
*Chlorella spp.* (16)	6	*Chlorella vulgaris*		3	2	1
		*Chlorella vulgaris isolate YSW04*	1	1		
		*Chlorella vulgaris KZN 23*	1			1
	7	*Chlorella sorokiniana*		2	1	
	8	*Chlorella ellipsoidea*	1			
	u	*Chlorella sp. (unidentified)*			2	
*Micractinium sp.* (15)	9	*Micractinium reisseri*	4	2		4
		*Micractinium reisseri RAIW01*	2			3
*Chlamydomonas spp.* (10)	10	*Chlamydomonas reinhardtii*	4	1		
	11	*Chlamydomonas incerta*	2	1		
	12	*Chlamydomonas peterfii*			1	
	u	*Chlamydomonas sp. (unidentified)*				1
*Fasciculochloris sp.* (3)	13	*Fasciculochloris boldii*		1		2
*Ankitrodesmus sp.* (2)	14	*Ankitrodesmus stipitatus*	1			1
*Ourococcus sp.* (2)	15	*Ourococcus multisporus*	1	1		
*Coelastrum sp.* (1)	16	*Coelastrum morum*			1	
*Desmodesmus sp.* (1)	17	*Desmodesmus sp. (unidentified)*		1		
*Ascochloris sp.* (1)	18	*Ascochloris multinucleata*	1			
*Asterarcys sp.* (1)	19	*Asterarcys quadricellulare*				1
*Parachlorella sp.* (1)	20	*Parachlorella beijerinckii*				1
*Chloromonas sp.* (1)	21	*Chloromonas oogama*				1
*Dictyosphaerium sp.* (1)	22	*Dictyosphaerium sp.(unidentified)*		1		
*Eudorina sp.* (1)	23	*Eudorina unicocca*				1
*Mychonastes sp.* (1)	24	*Mychonastes pushpae*				1
*Westella sp.* (1)	25	Westella botryoides				1
Shannon’s index *H* Evenness E_H_		2.8 0.6	1.0 0.3	0.7 0.2	0.4 0.2	1.2 0.4

u-Species unidentified.

**Table 2:** Location wise diversity of microalgal species.

Zone (genus richness per zone D)	Location codes*	Identification of microalgae (strain level)	Genera per location (genus diversity *H*)**
CZ (4.5)	AB	*Micractinium reisseri*	1
	BP	*Chlamydomonas reinhardtii* *Chlorella ellipsoidea* *Micractinium reisseri* *Scenedesmus obliquus isolate YSW14*	4 (1.29)
	BG	*Ankitrodesmus stipitatus Chlamydomonas incerta* *Chlorella vulgaris strain KZN 23 Micractinium reisseri Ourococcus multisporus* *Scenedesmus obliquus* *Scenedesmus obliquus isolate YSW14*	6 (1.69)
	BT	*Ascochloris multinucleata*	1
	HC	*Scenedesmus obliquus*	1
	KA	*Micractinium reisseri*	1
	LPR	*Chlamydomonas reinhardtii* *Scenedesmus obliquus* *Scenedesmus obliquus isolate YSW14*	2 (0.78)
	MR	*Chlamydomonas reinhardtii Scenedesmus obliquus isolate YSW14*	2 (0.78)
	MM	*Chlorella vulgaris YSW04*	1
	TP	*Micractinium reisseri isolate RAIW01*	1
	UPR	*Chlamydomonas incerta Chlamydomonas reinhardtii* *Micractinium reisseri isolate RAIW01* *Scenedesmus obliquus YSR02*	3 (1.05)
EZ (2.7)	AJ	*Chlorella vulgaris*	1
	BR	*Chlamydomonas peterfii* *Chlorella vulgaris isolate YSW04*	2 (0.78)
	CH	*Chlorella sorokiniana* *Chlorella* sp *Scenedesmus sp*	2 (0.78)
	EC	*Coelastrum morum*	1
	FCP	*Chlorella sp*	1
	PR	*Scenedesmus obliquus isolate YSW14*	1
NZ (3.3)	AP	*Scenedesmus obliquus*	1
	KR	*Chlorella vulgaris* *Scenedesmus obliquus*	2 (0.78)
	LSR	*Chlorella sorokiniana* *Chlorella vulgaris* *Chlorella vulgaris YSW04* *Micractinium reisseri*	2 (0.78)
	NP	*Fasciculochloris boldii*	1
	SE	*Chlorella vulgaris* *Dictyosphaerium sp* *Micractinium reisseri*	3 (1.05)
	USR	*Chlamydomonas incerta* *Chlamydomonas reinhardtii* *Chlorella sorokiniana* *Desmodesmus sp* *Ourococcus multisporus*	4 (1.29)
WZ (3.7)	BB	*Chloromonas oogama* *Scenedesmus obliquus isolate YSW14* *Scenedesmus obliquus YSR17*	2 (0.78)
	BP	*Chlorella vulgaris* *Micractinium reisseri* *Micractinium reisseri isolate RAIW01*	2 (0.78)
	CG	*Chlamydomonas sp* *Eudorina unicocca* *Fasciculochloris boldii* *Mychonastes pushpae* *Scenedesmus pectinatus*	5 (1.5)
	CL	*Ankitrodesmus stipitatus* *Fasciculochloris boldii* *Micractinium reisseri* *Micractinium reisseri isolate RAIW01* *Parachlorella beijerinckii* *Scenedesmus acuminatus* *Scenedesmus acutus* *Scenedesmus bajacalifornicus* *Scenedesmus obliquus* *Scenedesmus pectinatus* *Scenedesmus sp.* *Westella botryoides*	6 (1.69)
	JU	*Scenedesmus obliquus isolate YSW14*	1
	PC	*Chlorella vulgaris KZN 23* *Micractinium reisseri isolate RAIW01*	2 (0.78)
	Tu	*Asterarcys quadricellulare* *Micractinium reisseri*	2 (0.78)
	WCP	*Micractinium reisseri*	1

*Location laboratory codes.

**Diversity is calculated when there was only more than 1 isolate, D- Menhinick’s index, *H*-Shannon’s index.

## Discussion

The isolation, cultivation and identification of microalgae indigenous to an environment is primarily of ecological, biotechnological and commercial interest^1^. BBM, a traditional chemically defined medium was used to isolate microalgae in our study. We were able to successfully grow and isolate 17 different microalgal genera in this medium. We are aware that several genera may not be easily cultivable in this medium, hence we do not claim that this study is representative of the total microalgal biodiversity in Singapore.

While some microalgae were easy to identify based on cellular morphology, coccoid forms were generally difficult to distinguish based on microscopy. In this regard, species identification using LSU rDNA sequencing was a powerful technique. One of the greatest challenges was to standardize a method that would allow for extraction of DNA from different types of algae. This is probably because cell wall compositions of microalgae vary widely and may include cellulose, pectins, hemicelluloses, arabinogalactan proteins (AGPs), extensin, lignin, β-mannans, β-xylans, complex sulfated polysaccharides and glycoproteins^12^. Eventually, an in-house modification of the technique by Martin-Laurent *et al*^14^ led to a freeze-sonication based extraction method that was useful in extracting all the isolates we chose to study.

In addition to the algae reported in a past meta-analysis study from Singapore^4^ our study contributes the following 6 new species to existing reported genera *Ankitrodesmus stipitatus*, *Chlorella sorokiniana*, *Chlorella ellipsoidea*, *Micractinium reisseri*, *Scenedesmus pectinatus*, *Scenedesmus bajacalifornicus*. We also add the following new strains to the list- *Ascochloris multinucleate*, *Asterarcys quadricellulare*, *Chlamydomonas incerta*, *Chlamydomonas reinhardtii*, *Chlamydomonas peterfii*, *Parachlorella beijerinckii*, *Chloromonas oogama*, *Eudorina unicocca*, *Fasciculochloris boldii*, *Mychonastes pushpae*.

Microalgae are referred to as green gold because of their commercial value. They are cultivable throughout the year, have a low land demand and are a rich source of organic compounds. The three major uses of algae are biofuels (biochar, bioethanol, oil, biohydrogen), direct use (food and supplements for humans and animals), bioproducts (fatty acids, antioxidants, coloring agents, vitamins, anticancer and antimicrobial drugs)^1,21^. *Scenedesmus obliquus*, the most common microalga in our study, is reported to be used in effluent treatment, fish feed, biodiesel and pharmaceutical industries^22–25^, *Scenedesmus pectinatus, Scenedesmus acuminatus* and *Scenedesmus acutus* are also biofuel candidates^26–28^. *Scenedesmus bajacalifornicus* has pharmaceutical potential^29^. *Chlorella vulgaris* is widely used in nutrition and biodiesel^30^. Additional to these commercial uses, *Chlorella sorokiniana* has pharmaceutical and fish feed uses^31–33^. *Chlorella ellipsoidea* is additionally known to have pharmaceutical potential^34^. *Micractinium reisseri* is useful in waste water treatment^35^. *Chlamydomonas reinhardtii* is used as a molecular model and host organism for algal manipulation studies^36^, *Chlamydomonas incerta* is reported in pollutant removal^37^ and *Chlamydomonas peterfii* is used in chemical and radiation toxicity testing^38^. *Fasciculochloris boldii* and *Ourococcus multisporus* also have biofuel potential^39,40^. *Coelastrum sp.* and *Asterarcys quadricellulare* have nutrition and pharmaceutical potential^41,42^. *Parachlorella beijerinckii* is currently employed in the cosmetics industry^43^. *Chlorella* spp. and *Scenedesmus* spp. has high removal rates for nitrates, ammonia, nitrites and phosphates^44^. Due to the presence of poly unsatuarated fatty acids, *Ankitrodesmus, Chlorella, Chlamydomonas* and *Scenedesmus* spp are known to have cardioprotective value^45^. A large group of our microalgal genera have no prior biotechnological studies on them, such as *Desmodesmus sp.*, *Ascochloris sp.*, *Chloromonas sp.*, *Dictyosphaerium* sp., *Eudorina* sp. and *Mychonastes sp.* Future knowledge of this untapped potential could pave way for further scientific research.

Microalgae are unique to ecological sites of isolation. While our isolates were from fresh water; a past study on algae from bark in this city reported green algae *Dictyochloropsis* spp. and *Pseudomarvania aerophytica* among others which were not found in our aquatic sources^46^. *Scenedesmus obliquus* was the most common (27.5%) among our isolates. It was interesting to observe that studies on algal diversity in various countries showed different predominant genera. For example, studies from India showed *Oscillatoria* sp. and *Lynbygia* sp^47^; studies from South Africa showed *Chlorella* sp., *Neochloris* sp. and *Chlamydomonas* sp^48^; those from America showed *Chlorella* sp. and *Chlorococcum* sp.^49^ and studies from the Baltic showed *Synechococcus* and *Synechocystis* as most common^50^.

The presence of a wide variety of carbon-capturing photosynthetic microorganisms in the aqueous habitats interspersed through the high-rise city adds to the natural bio-diversity. Our study is important as it shares the most common microalgal genera present in inland fresh waters. Some of them are known to be of established commercial importance, while others are yet to be explored. We contribute additional knowledge of new microalgal genera and species. With the advent of algae occupying an important place in the pipeline of next generation fuels, foods and nutraceuticals, this study opens avenues for research in the biotechnology sector.
